# Long-term disease-free survival of an undifferentiated pleomorphic sarcoma of the spleen: A case report and literature review

**DOI:** 10.1097/MD.0000000000031642

**Published:** 2022-11-25

**Authors:** Atsushi Tomioka, Mitsuhiro Asakuma, Nao Kawaguchi, Koji Komeda, Tetsunosuke Shimizu, Kazuhisa Uchiyama, Sang-Woong Lee

**Affiliations:** a General and Gastroenterological Surgery, Osaka Medical and Pharmaceutical University, Takatsuki City, Osaka, Japan.

**Keywords:** case report, long-term survival, MFH, spleen, UPS

## Abstract

Undifferentiated pleomorphic sarcoma (UPS) primarily occurs in the soft tissues of the extremities, trunk, and retroperitoneum. As the primary UPS of the spleen (splenic UPS) is extremely rare, to the best of our knowledge, only 19 cases have been reported in English literature. No cases of long-term survival without a local or distant recurrence have been reported.

**Patient concerns:**

We report the case of a 37-year-old man who was referred to our hospital for a splenic tumor. He had no past medical or relevant familial history. On abdominal computed tomography (CT), a low attenuation solid mass and cystic component with mural calcifications were present at the lower pole of his spleen. The fluorodeoxyglucose-positron emission tomography (CT) indicated it as malignant tumor of the spleen.

**Diagnoses:**

The patient’s provisional diagnosis was deduced to be angiosarcoma, which was the most common malignant tumor of the spleen.

**Interventions:**

An elective laparoscopic splenectomy was performed, and the histology of the tumor was consistent with UPS (pT1, pN0, cM0, and AJCC^8th^). No adjuvant therapy was administered.

**Outcomes:**

Ten years have passed since the patient’s splenectomy, and he continues to do well, without evidence of local or distant recurrence.

**Lessons:**

To the best of our knowledge, this is the first case of long-term recurrence-free survival after surgical management of a splenic UPS. It is probable that radical splenectomy during the disease played the most important role in the patient’s long-term survival. Understanding the characteristic findings of a splenic UPS in an abdominal CT may help to diagnose properly.

## 1. Introduction

Primary undifferentiated pleomorphic sarcoma (UPS) is the most prevalent type of soft tissue sarcoma. It is usually encountered in the extremities and sometimes in retroperitoneum.^[1,2]^ The 5-year overall survival rate is 42% to 60%.^[3,4]^ However, UPS originated in the visceral organs is extremely rare and only a limited number of cases have been reported.^[5–11]^ Due to lack of appropriate follow-up data, its prognosis has still been unsure. UPS of spleen origin (splenic UPS) is also extremely rare and long-term survival case has never been reported. Herein, we report the case of a patient with a splenic UPS who is alive without a local or distant recurrence over 10 years after surgery, and survey of the literature for important points on this disease.

## 2. Case report

**Figure 1.**
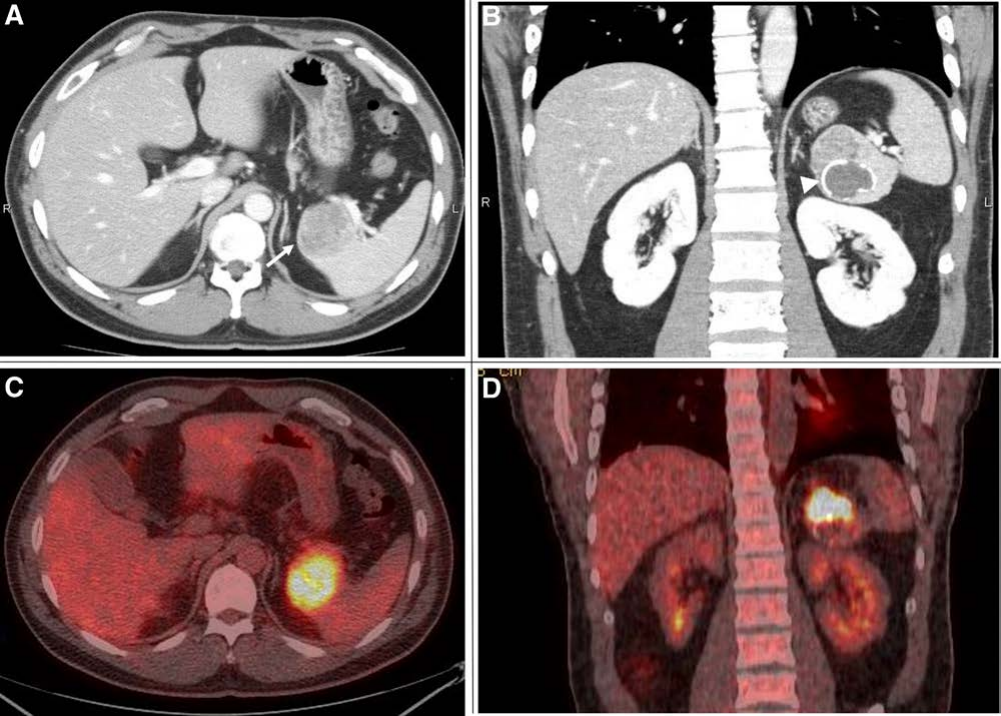
Preoperative CT and FDG-PET CT picture. (A) Computed tomography (CT) demonstrated a low attenuation solid mass protruding from the lower pole of the spleen (white arrow). (B) A cystic component with mural calcifications adjacent to the mass was shown. (arrow head). (C and D) The tumor was FDG avid on FDG-PET/CT. FDG-PET/CT = fluorodeoxyglucose-positron emission tomography.

**Figure 2.**
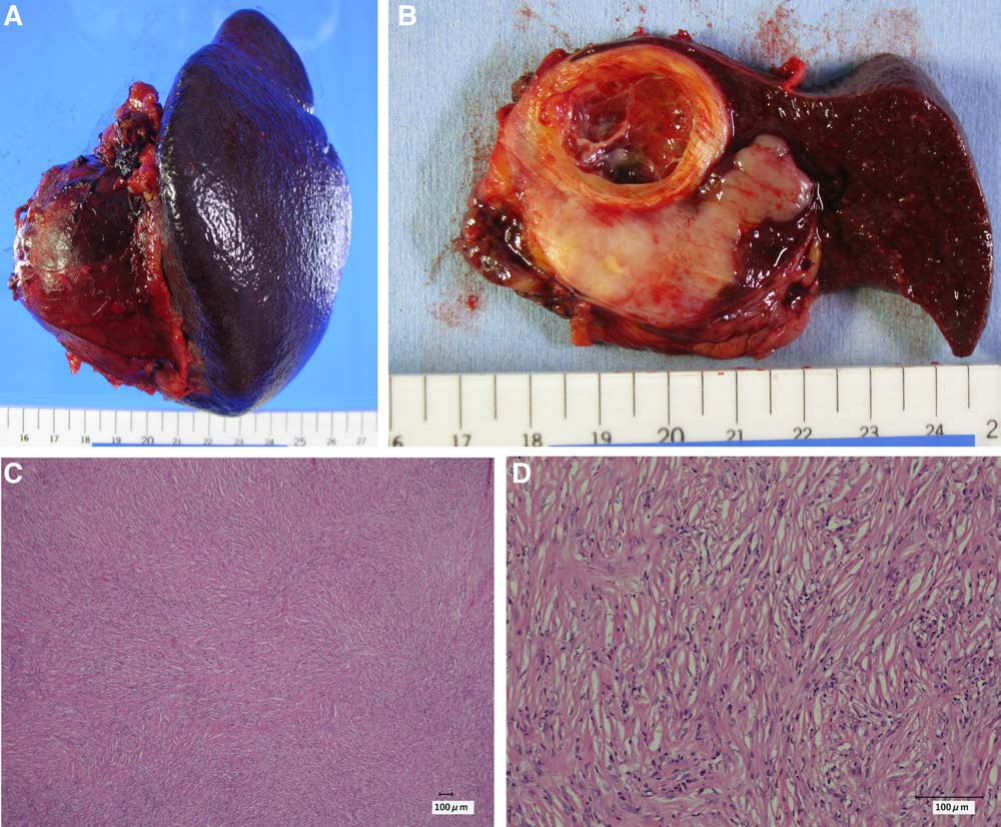
Specimen examination. (A and B) The tumor size was 5 × 5 × 4.5 cm and the large cystic component of the tumor contained necrotic and degenerative tissue. The tumor did not expose to splenic capsule and the surgical margin was negative. (C and D) Histologic examination of resected tissue samples. Hematoxylin and eosin (H&E) staining, original magnification ×40 (C), ×200 (D). It showed spindle-shaped cells arranged in a storiform pattern and accompanied by fibrous tissue.

A 37-year-old man was referred for evaluation of a splenic tumor that was detected on ultrasonography at an outside hospital a month before during a workup for chest pain. Although the cause of the patient’s chest pain remained unclear, it was self-limited. He did not have any past medical history, any drug allergy or relevant familial history. He had no history of alcohol intake and smoking. His abdomen was soft, nondistended, and nontender, without a palpable mass. His vital signs were normal. Clinical laboratory data were unremarkable: white blood cell count 5.49 × 10^3^/μL, hemoglobin 15.8 g/dL, platelet count 232 × 10^3^/μL, LDH 141U/L, CRP 0.25 mg/dL, CEA 2.8 ng/mL, CA19-9 39.3 U/mL and soluble IL-2 receptor 507 U/mL. Computed tomography (CT) demonstrated a low attenuation solid mass that measured approximately 4.5 × 5 × 6 cm protruding from the lower pole of the spleen. A cystic component with mural calcifications existed adjacent to the tumor. (Fig. 1A and B). The tumor was fluorodeoxyglucose (FDG) avid on fluorodeoxyglucose-positron emission tomography (FDG-PET/CT), and there was no evidence of distant metastases (Fig. 1C and D). The patient’s provisional diagnosis was angiosarcoma, which was the most common malignant tumor of the spleen. A biopsy was not performed because although it has been a common procedure recently, it was not a standard procedure in our facility at that time due to the risk of bleeding and peritoneal dissemination. As he did not require urgent treatment, an elective laparoscopic splenectomy was performed 10 days after the initial visit. Although the tumor was identified intraoperatively via careful inspection, it was not visible on the outside of the spleen. The entire spleen was resected without any rupture. The final tumor size was 5 × 5 × 4.5 cm (Fig. 2A and B). There were no findings of splenomegaly. Histopathological examination revealed that the large cystic component of the tumor contained necrotic and degenerative tissue. The tumor had not extended beyond the splenic capsule and the surgical margin was negative. Histology of the solid tumor showed spindle-shaped cells arranged in a storiform pattern and accompanied by fibrous tissue (Fig. 2C and D). There was inflammatory cell infiltration, most of which were monocytes. Immunohistochemistry was positive for vimentin, CD68, and α-1 antichymotrypsin and negative for CD31, D2-40, S-100, HHF25, and α-smooth muscle actin. The Ki-67 index was high. The patient’s definitive diagnosis was primary UPS of the spleen (pT1, pN0, cM0, and histological grade 2; AJCC^8th^). The patient was discharged uneventfully 9 days after surgery. He did not receive any adjuvant therapy. The postoperative surveillance protocol was below. An abdominal CT scan was performed every 6month up to 5 years after surgery and every 1 year from then until 10 years after surgery. He had no evidence of the disease at his 10-year follow-up visit.

## 3. Discussion

This case is, to our knowledge, the first of a long-term recurrence-free survival after a splenectomy for a splenic UPS. UPS, previously known as malignant fibrous histiocytoma (MFH), represents a heterogeneous group of sarcomas without a specific known line of differentiation. It is most frequently encountered as a malignant soft tissue tumor of the extremities, and primary UPS of the spleen is extremely rare.^[1,2]^ The first reported case of splenic UPS was termed a splenic MFH by Govoni in 1982. Only 19 total cases have been reported in English literature.^[12–28]^ Details of previous studies on splenic UPS, including the present work, are shown (Table 1). Fourteen of the (70.0%) patients were male, and 6 (30.0%) were female. The mean age was 54.3 years (range: 30–82 years). The most common symptoms of a splenic UPS were abdominal pain, weight loss, fever, and night sweats. The splenomegaly was seen in 5 cases (No.1,9,10,11,19). Splenic UPS is histologically highly cellular, has marked nuclear pleomorphism and abundant mitotic activity (including atypical forms and necrosis), and has areas of spindle cell morphology.^[29]^ Immunohistochemistry is often required to diagnose UPS. Although a specific antibody for UPS has not been identified, vimentin, CD68, α-SMA and α-antichymotorypsin were often positive in the previous studies (Table 1)^.[12–28]^ Immunohistochemistry may be useful for ruling out specific known lines of differentiation. Splenectomy is the most radical therapy. Although adjuvant radiotherapy had been well established to the UPS of soft tissue, unfortunately there was no strong evidence to splenic UPS. The principal purpose of adjuvant radiotherapy is to inactivate the microscopic extensions of tumor and histologically positive margins.^[3]^ In the case, the tumor hadn’t extended beyond the splenic capsule, and pathological R0 resection was performed. This was the reason why adjuvant radiotherapy was not performed. In 15 cases, including this case, a splenectomy was performed and a laparoscopic splenectomy was reported in 2 cases. In 2 cases, surgical treatment was not selected, because the diseases were too progressed. In the other 3 cases, we could not find descriptions about the way of treatment. Although the actual recurrence-free survival of surgically managed splenic UPS has not been reported, the longest individual recurrence-free survival of the cases currently available in the literature was 18 months. More than 10 years of long-term postoperative survival has never been reported. It may indicate that splenic UPS has an aggressive malignancy with a high potential for local recurrence and distant metastases. We reviewed all reported cases of splenic UPS to measure the primary tumor (T) category of each tumor if possible using the American Joint Committee on Cancer (AJCC) 8^th^ Edition (Table 2).^[30]^ Distant metastases at presentation were classified as M1. Prognostic staging has never been defined because there are limited data on UPS of the peritoneal cavity, including the spleen. We could evaluate the tumor status in 16 of 20 cases (80%). Three patients were T3 or T4 and M0 (18.8%) and 6 were M1 (37.5%); in 2 cases (No.14 and No.16), the tumor was ruptured (12.5%). Among them, 9 patients died within 19 months of surgery or diagnosis and 1 patient was alive with liver metastases 18 months after surgery (Case No. 3). The other 6 cases (37.5%) that were T1 or T2 and M0 were alive without recurrence at the time that their cases were published. These results may indicate that the reasons why splenic UPS is thought to have an extremely poor prognosis are that the majority of patients with splenic UPS have evidence of adjacent organ invasion or distant metastasis or that the tumor is ruptured when it is discovered. Conversely, if a splenic UPS is discovered early during the disease and resected radically, long-term survival might be expected. Unfortunately, in most cases, splenic tumors without symptoms tend to be discovered too late. Even if the tumor is discovered accidentally, judging whether the tumor is malignant or not is difficult. The specific CT characteristics of splenic UPS have not been well established. In this work, some characteristic CT findings were extracted. These were a low attenuation solid mass and a cystic component with mural calcifications adjacent to the mass, all of which were seen in this case (Fig 1A, B). Nine cases of splenic UPS cited in the literature also included CT findings. Calcifications were seen in 4 cases (44.4%) and ring like mural calcifications were seen in 3 (33.3%) (Table 1). Pseudocysts that contain hemorrhage and debris make up 80% of all splenic cystic lesions, and 50% of pseudocysts have mural calcification.^[31]^ As the histopathologic examination in this study revealed that the cystic component of this patient’s splenic UPS contained necrotic tissue, this cystic lesion resembles a splenic pseudocyst. In other words, splenic UPS might accompanies pseudocysts in the result of necrosis of tumor. Calcifications on CT have also been noted in the setting of an abdominal UPS in another literature.^[32]^ Of the 43 abdominal UPS discussed, 7 (16%) had intralesional calcifications on a preoperative abdominal CT, and ring like calcifications were seen in 3 cases (7.0%). The co-existence of a low attenuation solid mass and a cystic component with mural calcification may be an important hint to consider splenic UPS. Prior literature showed that UPS is FDG avid on FDG-PET/CT,^[33–35]^ which appears to apply to splenic UPS as well.^[27]^ CT findings as mentioned above may be useful to avoid delaying radical surgery early in the splenic UPS disease course.

**Table 1:** Summary of reported cases of splenic undifferentiated pleomorphic sarcoma.

Case no	Literature studies	Age/Gender	Tumor size (cm)	T and M value (AJCC 8th)	Synchronous metastasis	Clinical manifestation	CT findings	IHC staining (positive)	Treatment	Survival (after surgery)
1	1982, Govoni^[12]^	51/F	21 × 25 × 10	N/A	None	Abdominal pain, weight loss	N/A	N/A	Splenectomy	Alive at 7 months
2	1982, Wick^[13]^	54/M	N/A	N/A	None	N/A	N/A	N/A	N/A	Alive at 3 months
3		48/M	8	M1	Liver	N/A	N/A	N/A	Splenectomy Radiation	Alive at 18 months With liver metastasis
4		51/F	N/A	N/A	None	N/A	N/A	N/A	Splenectomy	Alive at 17 months
5	1988, Bruneton^[14]^	54/M	N/A	T3 (stomach, pancreas)	None	N/A	N/A	N/A	N/A	Died at 3 months
6	1990, Sieber^[15]^	41/M	N/A	M1	Omentum, Peritonium	N/A	Cystic component Calcification	N/A	Splenectomy	Died at 6 months
7	1993, Lieu^[16]^	71/M	N/A	M1	Liver	N/A	N/A	N/A	N/A	Died after surgery
8	1994, Bonilla^[17]^	42/F	N/A	N/A	Bone marrow	N/A	N/A	Vimentin, CD68	Splenectomy Radiation	Died at 8 months
9	1998, Mallpudi^[18]^	73/M	10	T3 (retroperitonium)	None	Fever, Night sweat Weight loss	N/A	N/A	Splenectomy	Died at 18th months
10	2001, Colovic^[19]^	45/F	11 × 10 × 7	T1/T2 M1	Liver	Abdominal pain, Weight loss Fever, Night sweat	N/A	Vimentin, CD68 HLADR, lysozyme, S-100	Splenectomy	Died at 15 months
11	2003, Ozaras^[20]^	51/F	12 × 11 × 10	T1/2	None	Abdominal pain, Weight loss Fever	Cystic component Mural calcification	N/A	Splenectomy	Not written
12	2006, Katsuura^[21]^	82/M	2.5 × 3	T2	None	Abdominal pain, Weight loss Fever	Low density mass	N/A	Splenectomy	Alive at 18 months
13	2010, Hashmi^[22]^	76/M	7.1 × 5.3	T1/T2	None	Abdominal pain	Cystic component	N/A	Lap-Splenectomy	Not reported
14	2011, He^[23]^	35/M	5 × 5	T1/T2 (rupture)	None	Abdominal pain	N/A	Vimentin, αSMA α1-antichymotrypsin	Splenectomy	Died at 7 months
15	2011, Ji-Feng^[24]^	48/M	5.2 × 4.6	T1/T2	None	Abdominal pain, Weight loss	Low density mass	Vimentin, CD68 α1-antichymotrypsin	Splenectomy	Alive at 13 months
16	2011, Amatya BM^[25]^	77/M	N/A	T3 M1 (rupture)	Renal hilum Adrenal grand Femoral bone marrow	Hemorrhagic shock	N/A	Vimentin, CD68	Without surgery	Died without surgery
17	2012, Dawson^[26]^	30/M	N/A	T1/T2	None	Abdominal pain	Solid mass Cystic component	Vimentin, CD68	Splenectomy	Not followed
18	2017, Makis W^[27]^	63/M	7.5 × 7.3 × 7	M1	Liver	Fever, Night sweat	Solid mass Peripheral enhancement	EBER, Fascin	Without surgery	Died at 16 months without surgery
19	2020, Ashmore^[28]^	56/F	15	T3 (diaphragm)	None	Abdominal pain, anemia	Cystic component Mural calcification	CD31	Splenectomy	Died at few months
20	2022, Tomioka (present case)	37/M	5 × 5 × 4.5	T1	None	Chest pain	Solid mass Cystic component Mural calcification	Vimentin, CD68 α1-antichymotrypsin	Lap-Splenectomy	Alive at 10 years

IHC = immunohistochemistry, N/A = information not available.

**Table 2:** The definition of TNM classification of soft tissue sarcoma in abdomen and thoracic visceral organs (AJCC 8th edition).

T category	T criteria	N category	N criteria	M category	M criteria
TX	Primary tumor cannot be assessed	N0	No regional lymph node metastasis or unknown lymph node status	M0	No distant metastasis
T1	Organ confined	N1	Regional lymph node metastasis	M1	Distant metastasis
T2	Tumor extension into tissue beyond organ				
T2a	Invades serosa or visceral peritoneum				
T2b	Extension beyond serosa (mesentery)				
T3	Invades another organ				
T4	Multifocal involvement				
T4a	Multifocal (2 sites)				
T4b	Multifocal (3–5 sites)				
T4c	Multifocal (>5 sites)				

AJCC = American Joint Committee on Cancer.

These days, the diagnostic accuracy and safety of percutaneous image-guided biopsy of the spleen was reported. If possible, splenic biopsy should be tried before surgery in the future.^[36]^Although splenic UPS might be considered as extremely malignant tumor, radical splenectomy early in the course (T1 or T2) may increase the likelihood of a good prognosis. To diagnose splenic UPS, some CT findings characterized in this work and FDG-PET/CT as well as fine needle biopsy might be useful. The prognostic feature and characteristic CT findings extracted in this work do not present strong evidence due to the small-size case series, which is a limitation of this work; however, we think these findings are worth the suggestion. This is the first case report of long-term recurrence-free survival following surgical treatment of a splenic UPS.

## Acknowledgments

The authors would like to thank Enago (www.enago.jp) for the English language review.

## Author contributions

**Conceptualization:** Tetsunosuke Shimizu.

**Data curation:** Koji Komeda.

**Investigation:** Nao Kawaguchi.

**Methodology:** Tetsunosuke Shimizu.

**Supervision:** Kazuhisa Uchiyama, Sang-Woong Lee.

**Visualization:** Nao Kawaguchi.

**Writing – original draft:** Atsushi Tomioka.

**Writing – review & editing:** Mitsuhiro Asakuma.
